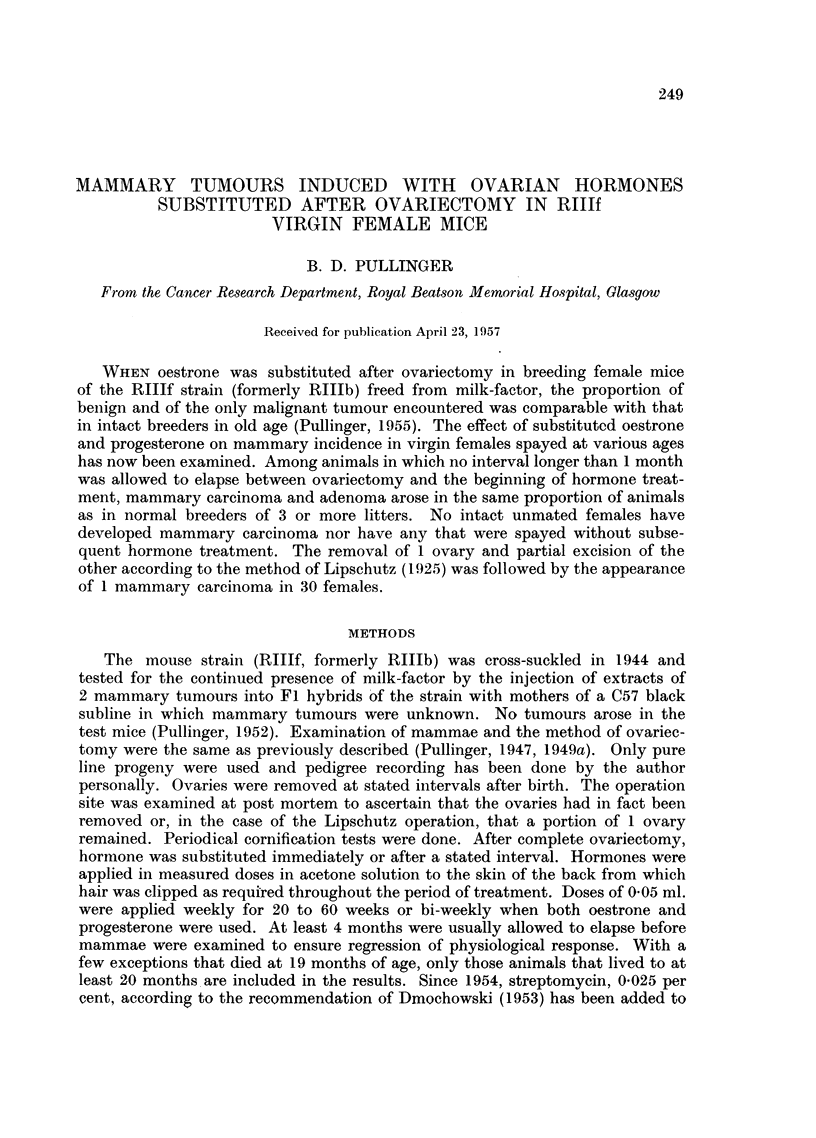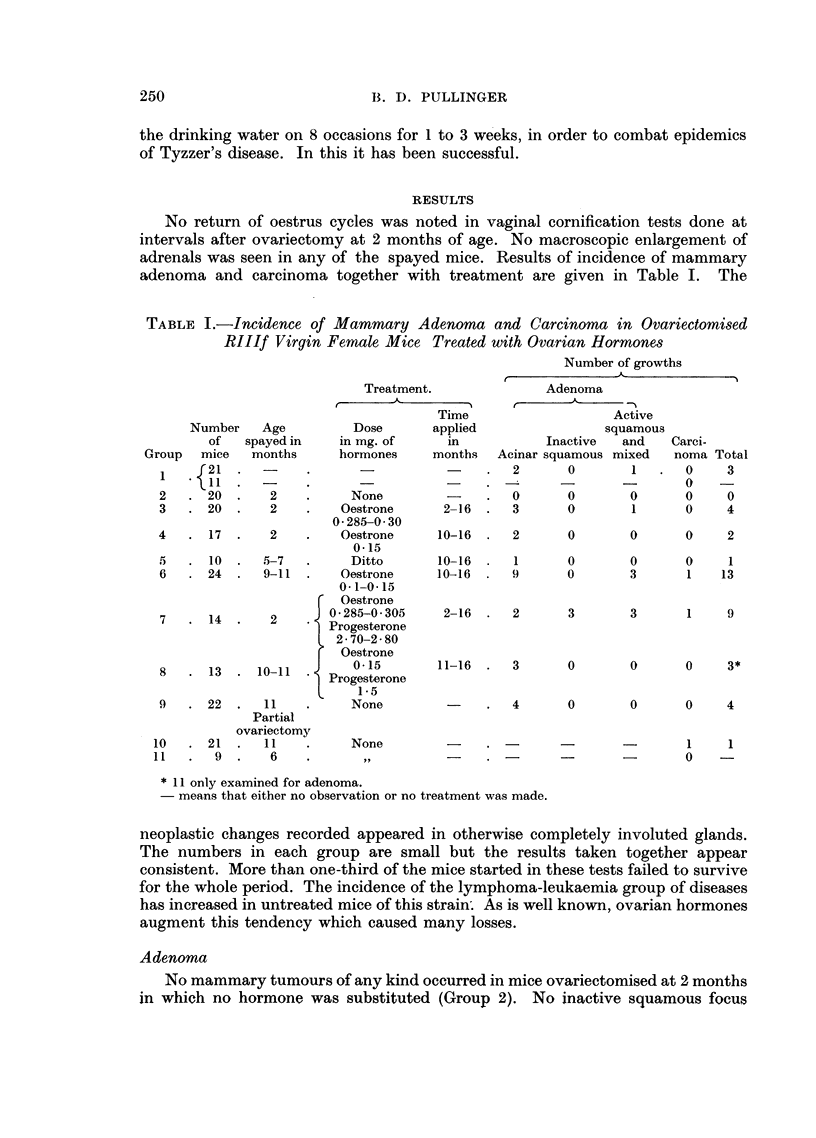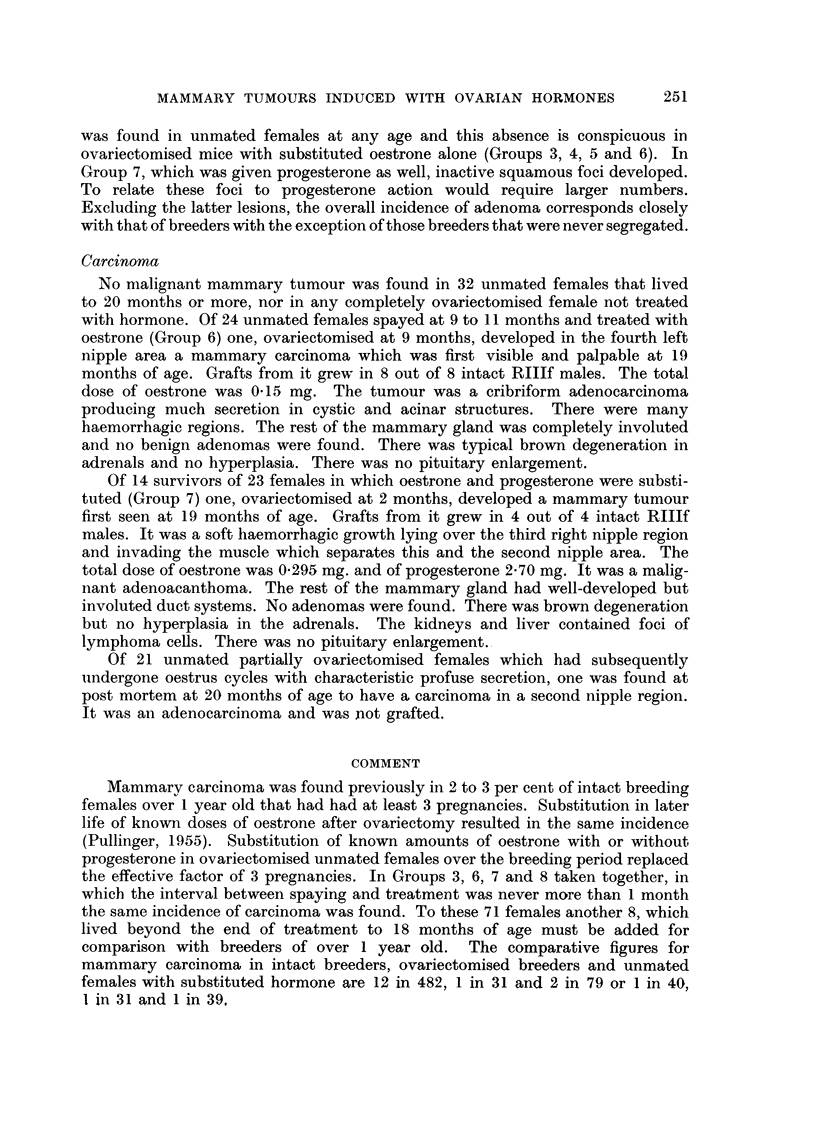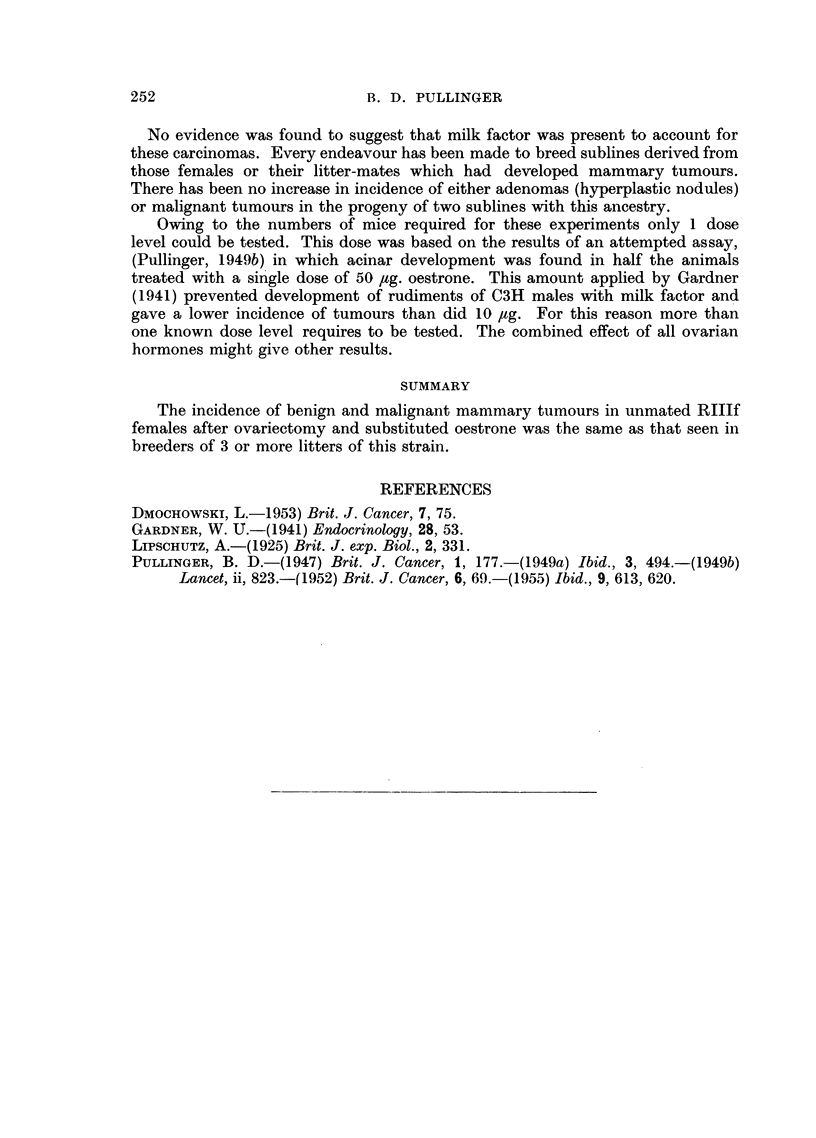# Mammary Tumours Induced with Ovarian Hormones Substituted after Ovariectomy in RIIIf Virgin Female Mice

**DOI:** 10.1038/bjc.1957.31

**Published:** 1957-06

**Authors:** B. D. Pullinger


### MAMMARY TUMOURS INDUCED WITH OVARIAN HORMONES SUBSTITUTED AFTER OVARIECTOMY IN RIIIf VIRGIN FEMALE MICE

B. D. PULLINGER

*From the Cancer Research Department, Royal Beatson Memorial Hospital, Glasgow*

Received for publication April 23, 1957

WHEN oestrone was substituted after ovariectomy in breeding female mice of the RIIIf strain (formerly RIIIb) freed from milk-factor, the proportion of benign and of the only malignant tumour encountered was comparable with that in intact breeders in old age (Pullinger, 1955). The effect of substituted oestrone and progesterone on mammary incidence in virgin females spayed at various ages has now been examined. Among animals in which no interval longer than 1 month was allowed to elapse between ovariectomy and the beginning of hormone treatment, mammary carcinoma and adenoma arose in the same proportion of animals as in normal breeders of 3 or more litters. No intact unmated females have developed mammary carcinoma nor have any that were spayed without subsequent hormone treatment. The removal of 1 ovary and partial excision of the other according to the method of Lipschutz (1925) was followed by the appearance of 1 mammary carcinoma in 30 females.

### METHODS

The mouse strain (RIIIf, formerly RIIIb) was cross-suckled in 1944 and tested for the continued presence of milk-factor by the injection of extracts of 2 mammary tumours into F1 hybrids of the strain with mothers of a C57 black subline in which mammary tumours were unknown. No tumours arose in the test mice (Pullinger, 1952). Examination of mammae and the method of ovariectomy were the same as previously described (Pullinger, 1947, 1949*a*). Only pure line progeny were used and pedigree recording has been done by the author personally. Ovaries were removed at stated intervals after birth. The operation site was examined at post mortem to ascertain that the ovaries had in fact been removed or, in the case of the Lipschutz operation, that a portion of 1 ovary remained. Periodical cornification tests were done. After complete ovariectomy, hormone was substituted immediately or after a stated interval. Hormones were applied in measured doses in acetone solution to the skin of the back from which hair was clipped as required throughout the period of treatment. Doses of 0·05 ml. were applied weekly for 20 to 60 weeks or bi-weekly when both oestrone and progesterone were used. At least 4 months were usually allowed to elapse before mammae were examined to ensure regression of physiological response. With a few exceptions that died at 19 months of age, only those animals that lived to at least 20 months are included in the results. Since 1954, streptomycin, 0·025 per cent, according to the recommendation of Dmochowski (1953) has been added to the drinking water on 8 occasions for 1 to 3 weeks, in order to combat epidemics of Tyzzer's disease. In this it has been successful.

### RESULTS

No return of oestrus cycles was noted in vaginal cornification tests done at intervals after ovariectomy at 2 months of age. No macroscopic enlargement of adrenals was seen in any of the spayed mice. Results of incidence of mammary adenoma and carcinoma together with treatment are given in Table I. The neoplastic changes recorded appeared in otherwise completely involuted glands. The numbers in each group are small but the results taken together appear consistent. More than one-third of the mice started in these tests failed to survive for the whole period. The incidence of the lymphoma-leukaemia group of diseases has increased in untreated mice of this strain. As is well known, ovarian hormones augment this tendency which caused many losses.

TABLE I.—*Incidence of Mammary Adenoma and Carcinoma in Ovariectomised RIIIf Virgin Female Mice Treated with Ovarian Hormones*

| | | | Treatment. | | Number of growths | | | | |
|---|---|---|---|---|---|---|---|---|---|
| | | | | | Adenoma | | | | |
| Group | Number of mice | Age spayed in months | Dose in mg. of hormones | Time applied in months | Acinar | Inactive squamous | Active squamous and mixed | Carci-noma | Total |
| 1 | 21 | — | — | — | 2 | 0 | 1 | 0 | 3 |
| | 11 | — | — | — | — | — | — | 0 | — |
| 2 | 20 | 2 | None | — | 0 | 0 | 0 | 0 | 0 |
| 3 | 20 | 2 | Oestrone 0·285–0·30 | 2–16 | 3 | 0 | 1 | 0 | 4 |
| 4 | 17 | 2 | Oestrone 0·15 | 10–16 | 2 | 0 | 0 | 0 | 2 |
| 5 | 10 | 5–7 | Ditto | 10–16 | 1 | 0 | 0 | 0 | 1 |
| 6 | 24 | 9–11 | Oestrone 0·1–0·15 | 10–16 | 9 | 0 | 3 | 1 | 13 |
| 7 | 14 | 2 | Oestrone 0·285–0·305 Progesterone 2·70–2·80 | 2–16 | 2 | 3 | 3 | 1 | 9 |
| 8 | 13 | 10–11 | Oestrone 0·15 Progesterone 1·5 | 11–16 | 3 | 0 | 0 | 0 | 3* |
| 9 | 22 | 11 Partial ovariectomy | None | — | 4 | 0 | 0 | 0 | 4 |
| 10 | 21 | 11 | None | — | — | — | — | 1 | 1 |
| 11 | 9 | 6 | ,, | — | — | — | — | 0 | — |

* 11 only examined for adenoma.
— means that either no observation or no treatment was made.

### *Adenoma*

No mammary tumours of any kind occurred in mice ovariectomised at 2 months in which no hormone was substituted (Group 2). No inactive squamous focus was found in unmated females at any age and this absence is conspicuous in ovariectomised mice with substituted oestrone alone (Groups 3, 4, 5 and 6). In Group 7, which was given progesterone as well, inactive squamous foci developed. To relate these foci to progesterone action would require larger numbers. Excluding the latter lesions, the overall incidence of adenoma corresponds closely with that of breeders with the exception of those breeders that were never segregated.

*Carcinoma*

No malignant mammary tumour was found in 32 unmated females that lived to 20 months or more, nor in any completely ovariectomised female not treated with hormone. Of 24 unmated females spayed at 9 to 11 months and treated with oestrone (Group 6) one, ovariectomised at 9 months, developed in the fourth left nipple area a mammary carcinoma which was first visible and palpable at 19 months of age. Grafts from it grew in 8 out of 8 intact RIIIf males. The total dose of oestrone was 0·15 mg. The tumour was a cribriform adenocarcinoma producing much secretion in cystic and acinar structures. There were many haemorrhagic regions. The rest of the mammary gland was completely involuted and no benign adenomas were found. There was typical brown degeneration in adrenals and no hyperplasia. There was no pituitary enlargement.

Of 14 survivors of 23 females in which oestrone and progesterone were substituted (Group 7) one, ovariectomised at 2 months, developed a mammary tumour first seen at 19 months of age. Grafts from it grew in 4 out of 4 intact RIIIf males. It was a soft haemorrhagic growth lying over the third right nipple region and invading the muscle which separates this and the second nipple area. The total dose of oestrone was 0·295 mg. and of progesterone 2·70 mg. It was a malignant adenoacanthoma. The rest of the mammary gland had well-developed but involuted duct systems. No adenomas were found. There was brown degeneration but no hyperplasia in the adrenals. The kidneys and liver contained foci of lymphoma cells. There was no pituitary enlargement.

Of 21 unmated partially ovariectomised females which had subsequently undergone oestrus cycles with characteristic profuse secretion, one was found at post mortem at 20 months of age to have a carcinoma in a second nipple region. It was an adenocarcinoma and was not grafted.

### COMMENT

Mammary carcinoma was found previously in 2 to 3 per cent of intact breeding females over 1 year old that had had at least 3 pregnancies. Substitution in later life of known doses of oestrone after ovariectomy resulted in the same incidence (Pullinger, 1955). Substitution of known amounts of oestrone with or without progesterone in ovariectomised unmated females over the breeding period replaced the effective factor of 3 pregnancies. In Groups 3, 6, 7 and 8 taken together, in which the interval between spaying and treatment was never more than 1 month the same incidence of carcinoma was found. To these 71 females another 8, which lived beyond the end of treatment to 18 months of age must be added for comparison with breeders of over 1 year old. The comparative figures for mammary carcinoma in intact breeders, ovariectomised breeders and unmated females with substituted hormone are 12 in 482, 1 in 31 and 2 in 79 or 1 in 40, 1 in 31 and 1 in 39.

No evidence was found to suggest that milk factor was present to account for these carcinomas. Every endeavour has been made to breed sublines derived from those females or their litter-mates which had developed mammary tumours. There has been no increase in incidence of either adenomas (hyperplastic nodules) or malignant tumours in the progeny of two sublines with this ancestry.

Owing to the numbers of mice required for these experiments only 1 dose level could be tested. This dose was based on the results of an attempted assay, (Pullinger, 1949*b*) in which acinar development was found in half the animals treated with a single dose of 50 $\mu$g. oestrone. This amount applied by Gardner (1941) prevented development of rudiments of C3H males with milk factor and gave a lower incidence of tumours than did 10 $\mu$g. For this reason more than one known dose level requires to be tested. The combined effect of all ovarian hormones might give other results.

### SUMMARY

The incidence of benign and malignant mammary tumours in unmated RIIIf females after ovariectomy and substituted oestrone was the same as that seen in breeders of 3 or more litters of this strain.

### REFERENCES

DMOCHOWSKI, L.—1953) *Brit. J. Cancer*, **7**, 75.
GARDNER, W. U.—(1941) *Endocrinology*, **28**, 53.
LIPSCHUTZ, A.—(1925) *Brit. J. exp. Biol.*, **2**, 331.
PULLINGER, B. D.—(1947) *Brit. J. Cancer*, **1**, 177.—(1949*a*) *Ibid.*, **3**, 494.—(1949*b*) *Lancet*, ii, 823.—(1952) *Brit. J. Cancer*, **6**, 69.—(1955) *Ibid.*, **9**, 613, 620.